# Novel bi-allelic *MSH4* variants causes meiotic arrest and non-obstructive azoospermia

**DOI:** 10.1186/s12958-022-00900-x

**Published:** 2022-01-28

**Authors:** Peng Li, Zhiyong Ji, Erlei Zhi, Yuxiang Zhang, Sha Han, Liangyu Zhao, Ruhui Tian, Huixing Chen, Yuhua Huang, Jing Zhang, Huirong Chen, Fujun Zhao, Zhi Zhou, Zheng Li, Chencheng Yao

**Affiliations:** 1 Department of Andrology, Center for Men’s Health, Department of ART, Institute of Urology, Urologic Medical Center, Shanghai General Hospital, Shanghai Jiao Tong University School of Medicine, Shanghai, 200080 China; 2 State Key Laboratory of Reproductive Medicine, Nanjing Medical University, Nanjing, 211116 China; 3 The Reproductive Medicine Research Center, The Sixth Affiliated Hospital of Sun Yat-sen University, Guangzhou, 510620 China; 4 School of Life Science and Technology, ShanghaiTech University, Shanghai, 201210 China

**Keywords:** Non-obstructive azoospermia, Meiosis, *MSH4*, Male infertility

## Abstract

**Background:**

Non-obstructive azoospermia (NOA) is one of the most severe type in male infertility, and the genetic causes of NOA with meiotic arrest remain elusive.

**Methods:**

Four Chinese families with NOA participated in the study. We performed whole-exome sequencing (WES) for the four NOA-affected patients in four pedigrees. The candidate causative gene was further verified by Sanger sequencing. Hematoxylin and eosin staining (H&E) and immunohistochemistry (IHC) were carried out to evaluate the stage of spermatogenesis arrested in the patients with NOA.

**Results:**

We identified two novel homozygous frameshift mutations of *MSH4* and two novel compound heterozygous variants in *MSH4* in four pedigrees with NOA. Homozygous loss of function (LoF) variants in *MSH4* was identified in the NOA-affected patient (P9359) in a consanguineous Chinese family (NM_002440.4: c.805_812del: p.V269Qfs*15) and one patient with NOA (P21504) in another Chinese family (NM_002440.4: c.2220_2223del:p.K741Rfs*2). Also, compound heterozygous variants in *MSH4* were identified in two NOA-affected siblings (P9517 and P9517B) (NM_002440.4: c.G1950A: p.W650X and c.2179delG: p.D727Mfs*11), and the patient with NOA (P9540) (NM_002440.4: c.G244A: p.G82S and c.670delT: p.L224Cfs*3). Histological analysis demonstrated lack of spermatozoa in seminiferous tubules of all patients and IHC showed the spermatogenesis arrested at the meiotic prophase I stage. Consistent with the autosomal recessive mode of inheritance, all of these mutations were inherited from heterozygous parental carriers.

**Conclusions:**

We identified that six novel mutations in *MSH4* responsible for meiotic arrest and NOA. And these results provide researchers with a new insight to understand the genetic etiology of NOA and to identify new loci for genetic counselling of NOA.

**Supplementary Information:**

The online version contains supplementary material available at 10.1186/s12958-022-00900-x.

## Introduction

Male infertility is a multifactorial heterogeneous pathological condition, affecting ∼7% of male general population. Non-obstructive azoospermia (NOA) is one of the most severe male reproductive diseases, which occurs in ∼1% of men [1]. NOA displays germ cell absence or reduction owing to the testicular atrophy, and NOA usually results from Y chromosome microdeletion, chromosome abnormalities, hypogonadism, varicocele, testicular tumor, and improper drug administration. However, 70% of patients with NOA were idiopathic, the etiology and pathology of NOA remains unclear.

Meiosis is an intricate process during the spermatogenesis, including one round of DNA replication and two rounds of chromosome segregation. The defect of meiosis could lead to meiotic arrest and NOA. During the meiosis prophase I, homologous recombination resulted in crossover (CO) formation in the context of the synaptonemal complex (SC). It’s initiated by formation of DNA double-strand breaks via catalyzation of SPO11 [2]. And the recombination intermediates are repaired towards either CO or non-crossover (NCO) pathways [3]. The heterodimer of MutL (MLH1 and MLH3) is responsible for formation of the major COs [4–7], and MutS functions to recruit MutL to the SC during pachynema. Unlike MSH2-MSH6 (MutSα) and MSH2-MSH3 (MutSβ) heterodimers, which are involved in mismatch repair during mitosis, MSH4-MSH5 (MutSγ) functions exclusively during meiotic prophase I [8]. It has been demonstrated that loss of *Msh4* or *Msh5* results in defects of prophase I progression in mice, with almost complete failure of homologous synapsis, and cell death prior to pachynema [9–11]. Also, up to now, whole-exome sequencing (WES) of pedigree studies reported that only two types of homozygous mutation in *MSH4* (NM_002440.4: c.2261C > T and c.1552C > T) were associated with NOA [12, 13]. Thus, many other types of mutation in *MSH4* which were associated with NOA remain to be elucidated.

Here, we identified six novel mutations in the meiotic gene *MSH4* in four pedigrees with NOA. Two homozygous frameshift mutations were identified in two families. Also, two compound heterozygous mutations in *MSH4* were reported in two other pedigrees with NOA.

## Materials and methods

### Study approval

This study was approved by the Institutional Ethical Review Committee of Shanghai General Hospital, Shanghai Jiao Tong University (2020SQ199), and the informed consent of clinical data and testicular tissue for research was obtained from the donors.

### Study subjects

Four families with NOA were enrolled in this study, and all the NOA-affected patients had no known causal factors for male infertility, including varicocele, radiation, chemotherapy, orchitis, cryptorchidism, and testicular cancer. The family histories were collected. Semen analysis and sexual hormones examination were performed in all the NOA-affected patients. Also, the genetic evaluation including karyotype, Y chromosome microdeletions, and WES were conducted in all the patients with NOA. Furthermore, routine testicular biopsy was performed for histopathological examination.

### WES

The genomic DNA was extracted from the blood of the four probands using the TIANamp Blood DNA Kit (TIANGEN) according to the manufacturer’s instructions. DNA was fragmented through Covaris focused ultrasonication. Known exons and exon–intron boundary sequences were captured using xGen® Exome Research Panel (IDT, USA), and sequencing DNA libraries were prepared following the manufacturer’s instruction. Sequencing was performed on an Illumina HiSeq × 10 platform. Sequencing reads were aligned to the human genome (GRCh37/hg19) using Burrows–Wheeler Aligner. Both single-nucleotide variants and indels within the captured coding exonic intervals were called using GATK, Platypus, VarScan, LoFreq, FreeBayes, SNVer, SAMtools and VarDict. Also, the variants were filtered and annotated using the ANNOVAR software. Because autosomal recessive or X-linked inheritance were assumed for meiotic defect, genes with two alleles of potentially deleterious missense mutations (SIFT, PolyPhen-2 and Mutation Taster), or LoF mutations were kept for further analysis. Moreover, we compared candidate genes with human germline-enriched genes in the database and known pathogenic genes for azoospermia in mice. The aforementioned sequencing and bioinformatic analyses were conducted together with the Nuprobe company (Shanghai, China). The datasets used and analyzed during the current study are available from the corresponding author on reasonable request.

### Sanger sequencing

Polymerase chain reaction (PCR) was performed in four families with NOA. The primers were shown in supplementary Table S1. And the PCR products were bidirectionally sequenced by Sanger sequencing using a 3730xl DNA Analyzer (Applied Biosystems, Forster City, California, USA).

### H&E staining

The testicular tissues from the NOA-affected probands were fixed in 4% paraformaldehyde solution overnight, embedded in paraffin and sectioned at 5 μm thickness. Paraffin sections were dewaxed with xylene for 30 min, and then, rehydrated sequentially in 95, 90, 85 and 70% ethanol each for 10 min, followed in PBS solution for 10 min, and stained with hematoxylin and eosin according to standard protocols (catalogue number: ab245880, Abcam, Cambridge, UK). All the staining sections were captured by phase-contrast microscope to observe the structure changes of testicular tissues (Leica).

### IHC

The testicular biopsies were obtained from the four patients with NOA and the OA-affected patient as the positive control. The testicular tissue was fixed overnight in 4% paraformaldehyde at 4 °C, and then embedded in warm paraffin (60 °C). The biopsies were sectioned at 5 μm thickness. The tissue sections were dewaxed in xylene, re-hydrated in a descending alcohol gradient, and heated in sodium citrate buffer (90–98 °C) for 15 min for antigen retrieval. After blocking with 5% BSA for 1 h at room temperature, the sections were incubated overnight with anti-SYCP3 (dilution: 1:25; catalogue number: AF3750, R&D Systems), anti-γH2AX (dilution: 1:300; catalogue number: 2668445, Millipore), anti-DMC1 (dilution: 1:100; catalogue number: sc-373,862, Santa Cruz) and PNA (dilution: 1:400; catalogue number: L21409, Thermo Fisher Scientific) at 4 °C. The sections were washed thrice with PBS-T (Phosphate buffer saline-Tween), and incubated with highly cross-adsorbed secondary antibody conjugated with Alexa Fluor® 488 or Alexa Fluor® 594 (dilution: 1:400; Thermo Fisher Scientific) for 1 h at room temperature. The sections were washed three times with PBS-T and counterstained with 4′,6-diamidino-2-phenylindole to label the nuclei. The images were captured by fluorescence microscope (Leica).

## Results

### Clinical findings

**Fig. 1:**
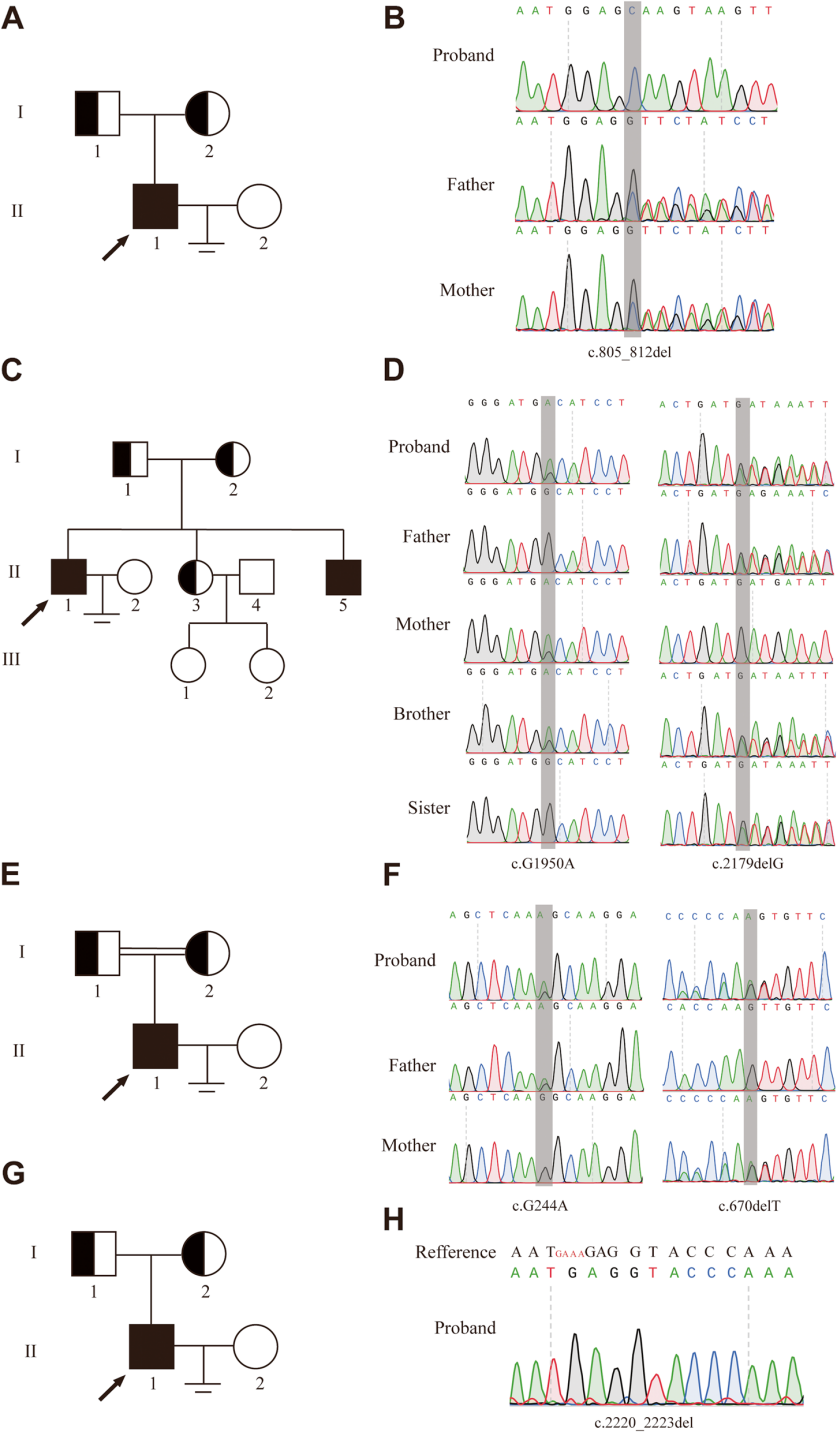
Bi-allelic mutations in *MSH4* identified in patients with NOA. **A**, **C**, **E**, and **G** Pedigree of NOA-affected families (P9359, P9517, P9540 and P21504). WES was performed in the patients with NOA (indicated by arrowheads), and the genotypes of the probands and other family members were examined by Sanger sequencing (**B**, **D**, **F**, and **H**)

Four Chinese families with infertility participated in the present study (Fig. 1). All the probands had a history of male infertility for several years, and routine semen analyses revealed complete azoospermia with normal volume. They were in good physical condition. The testes were palpable and bilateral testicular size was normal, without varicocele. Laboratory examination showed that sex hormone levels in the four patients were comparable to the reference values. They had normal 46, XY karyotypes and no Y chromosome microdeletions. In family P9540, the parents of the proband were married first cousins, however, there was no family histories of consanguinity in other three families. The probands in four families underwent the testicular sperm extraction procedure in Shanghai General Hospital, and histopathological analysis revealed meiotic arrest in the four patients with NOA. Collectively, all the clinical characteristics of the NOA-affected patients were summarized in Table 1.

**Table 1:** Clinical characteristics of the patients with bi-allelic variants of *MSH4* at the clinical assessment

Subject	P9359	P9517-1	P9517-2	P9540	P21504
Secondary sexual characteristics	Normal	Normal	Normal	Normal	Normal
Testicular volume (Left/Right, ml)	12/12	12/12	/	12/12	15/15
Karyotype	46,XY	46,XY	/	46,XY	46,XY
Y Chromosome microdeletions	No	No	/	No	No
Follicle-stimulating hormone (IU/L)	4.4	9.66	/	10.83	4.7
Luteinizing hormone (IU/L)	5.54	5.52	/	5.44	3.4
Testosterone (nmol/L)	3.45	3.95	/	5.63	2.87

### Identification of the *MSH4* mutations in four pedigrees with NOA

WES was performed on the probands in the four families. After the genetic analyses pipeline, homozygous LoF variant in *MSH4* (family P9359 (NM_002440.4: c.805_812del:p.V269Qfs*15); family P21504 (NM_002440.4: c.2220_2223del:p.K741Rfs*2), and compound heterozygous mutations in *MSH4* (P9517 (NM_002440.4: c.G1950A:p.W650X and c.2179delG:p.D727Mfs*11; P9540 (NM_002440.4: c.G244A:p.G82S and c.670delT:p.L224Cfs*3)) were assumed as the most likely causes of meiosis defect in the four patients with NOA, respectively (Table 2).

**Table 2:** In silico analysis of the novel variants of *MSH4*

Patient No.	Change in coding DNA (NM_001003811)	Protein/RNA change	Phenotype	GnomAD minor allele frequency (%)	ExAC	Polyphen2	SIFT	Mutation taster	CADD
P9359	c.805_812del	p.V269Qfs*15	MA	NA	NA	NA	NA	NA	NA
P9517	c.G1950A	p.W650X	MA	1.2 × 10^−5^	8.3 × 10^−6^	NA	NA	disease_causing_automatic	NA
P9517	c.2179delG	p.D727Mfs*11	MA	NA	NA	NA	NA	NA	NA
P9540	c.G244A	p.G82S	MA	NA	NA	Benign	Deleterious	Deleterious	NA
P9540	c.670delT	p.L224Cfs*3	MA	NA	NA	NA	NA	NA	NA
P21504	c.2220_2223del	p.K741Rfs*2	MA	NA	NA	NA	NA	NA	NA

*Abbreviations*: *MA* Maturation Arrest, *NA* Not Available

**Fig. 2:**
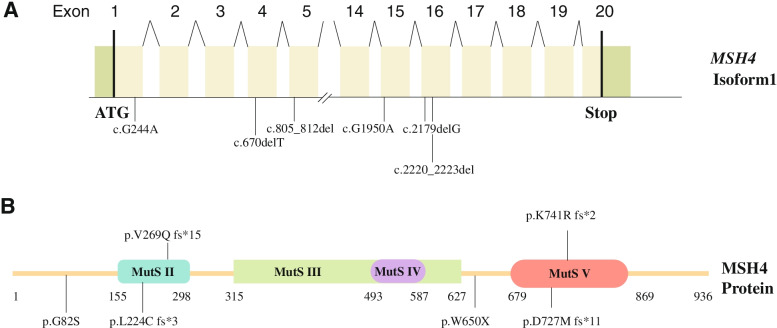
The location of six novel variants in *MSH4*. The genomic structure of *MSH4*, with variants mapped to isoform 1 (GenBank accession number, NM_002440.4). **A **Green rectangles represent noncoding exons, and yellow rectangles represent coding exons. The coding sequence of the gene begins at nucleotides that encode a start codon in exon 1 and ends in exon 20 at a stop codon. All variants detected in men with NOA are shown. **B** The predicted MSH4 domains with MutS domain II, III, IV, V, containing regions (amino acid positions 155–298, 315-627, 493-587, 679-869). Variants (black lines) are indicated in predicted location

Sanger sequencing was then performed to validate the results in WES sequencing using DNA samples from the family members. In family P9359, homozygous LoF mutation in *MSH4* (NM_002440.4: c.805_812del:p.V269Qfs*15) was identified in the proband (Fig. 1A, B), and this frameshift variant resulted in a truncated MSH4 protein without expression of MutS III-V domain. In family P21504, homozygous LoF mutations in *MSH4* (NM_002440.4: c.2220_2223del:p.K741Rfs*2) also led to a truncated MSH4 protein with the defect in expression of MutS V domain. Consistent with an autosomal recessive mode of inheritance, the unaffected parents were heterozygous carriers of this same *MSH4* variant (Fig. 1G, H). In family P9517, compound heterozygous LoF mutations in *MSH4* (NM_002440.4: c.G1950A:p.W650X and c.2179delG: p.D727Mfs*11) were identified in the proband and his brother (Fig. 1C, D). A maternally inherited nonsense variant (NM_002440.4: c.G1950A:p.W650X) resulted in a premature stop, and a paternally inherited frameshift variant (NM_002440.4: c.2179delG: p.D727Mfs*11) led to a truncated MSH4 protein. These two variants resulted in the defect in expression of MutS V domain. Furthermore, the proband’s sister, who carried heterozygous paternal inherited frameshift variant (NM_002440.4: c.2179delG:p.D727Mfs*11) has two daughters, suggesting that compound heterozygous LoF mutations in *MSH4* were the cause of NOA in the two brothers. In family P9540, a maternally inherited frameshift variant (NM_002440.4: c.670delT:p.L224Cfs*3) led to a truncated MSH4 protein without expression of MutS III-V domain and a paternally inherited variant (NM_002440.4: c.G244A: p.G82S) resulted in transition of Glycine to Serine at the position 82 of MSH4. And it showed strong evolutionary conservation at this position, and this missense variant was predicted to be potentially deleterious simultaneously by SIFT and MutationTaster. Altogether, two homozygous LoF mutations and two compound heterozygous mutations in *MSH4* were identified in four patients with NOA (Fig. 2).

### Meiotic arrest phenotype in the patients with bi-allelic mutations in *MSH4*

**Fig. 3:**
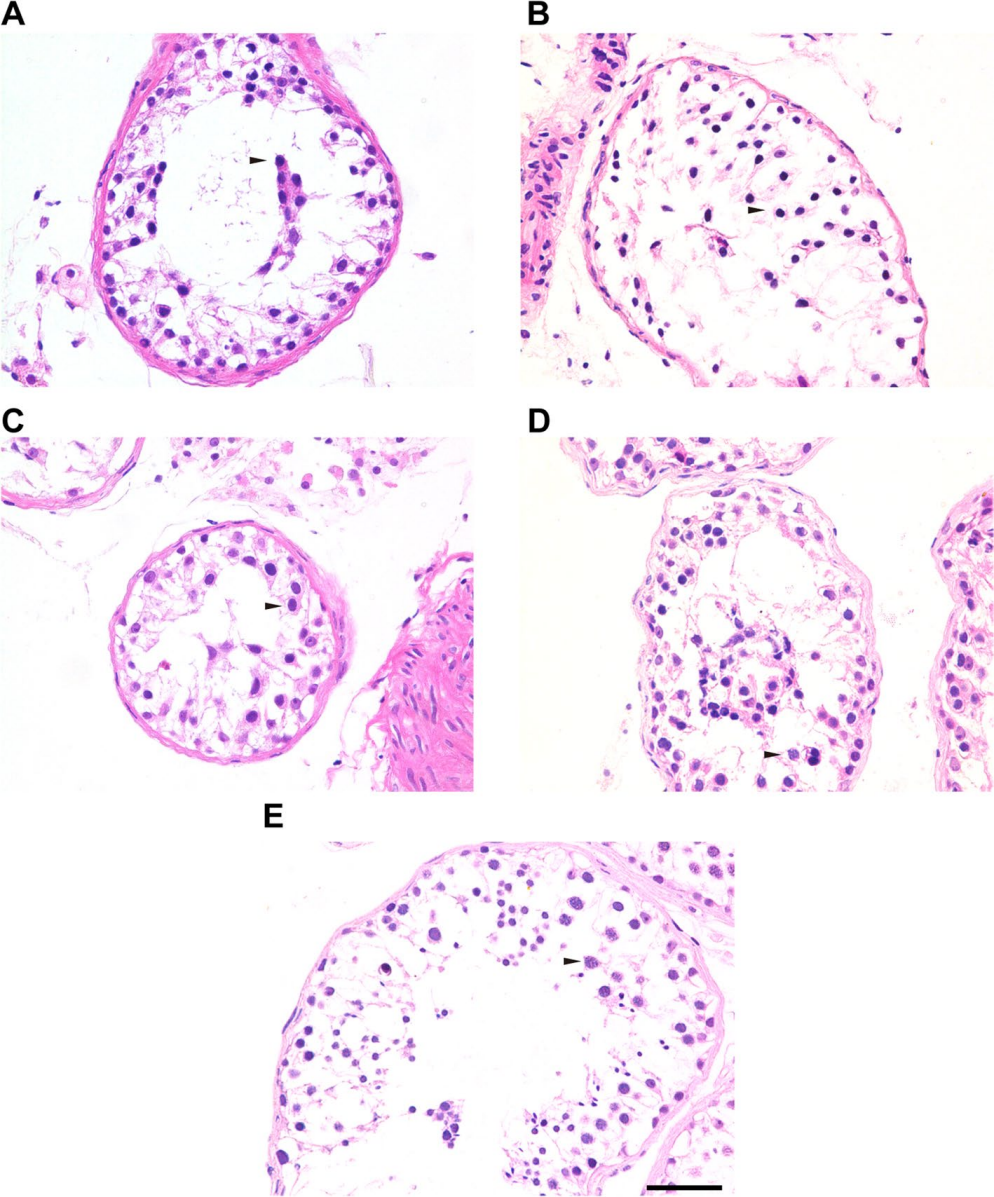
H&E staining of cross-sections in NOA-affected patients and a patient with OA as positive control. **A** H&E staining of cross-sections of testicular biopsy from the patient P9359 (**A**), P9517(**B**), the P9540 (**C**), P21504 (**D**) and a patient with OA as the positive control (**E**). Black triangles indicate the spermatocytes in the testis. Scale bars = 50 μm

**Fig. 4:**
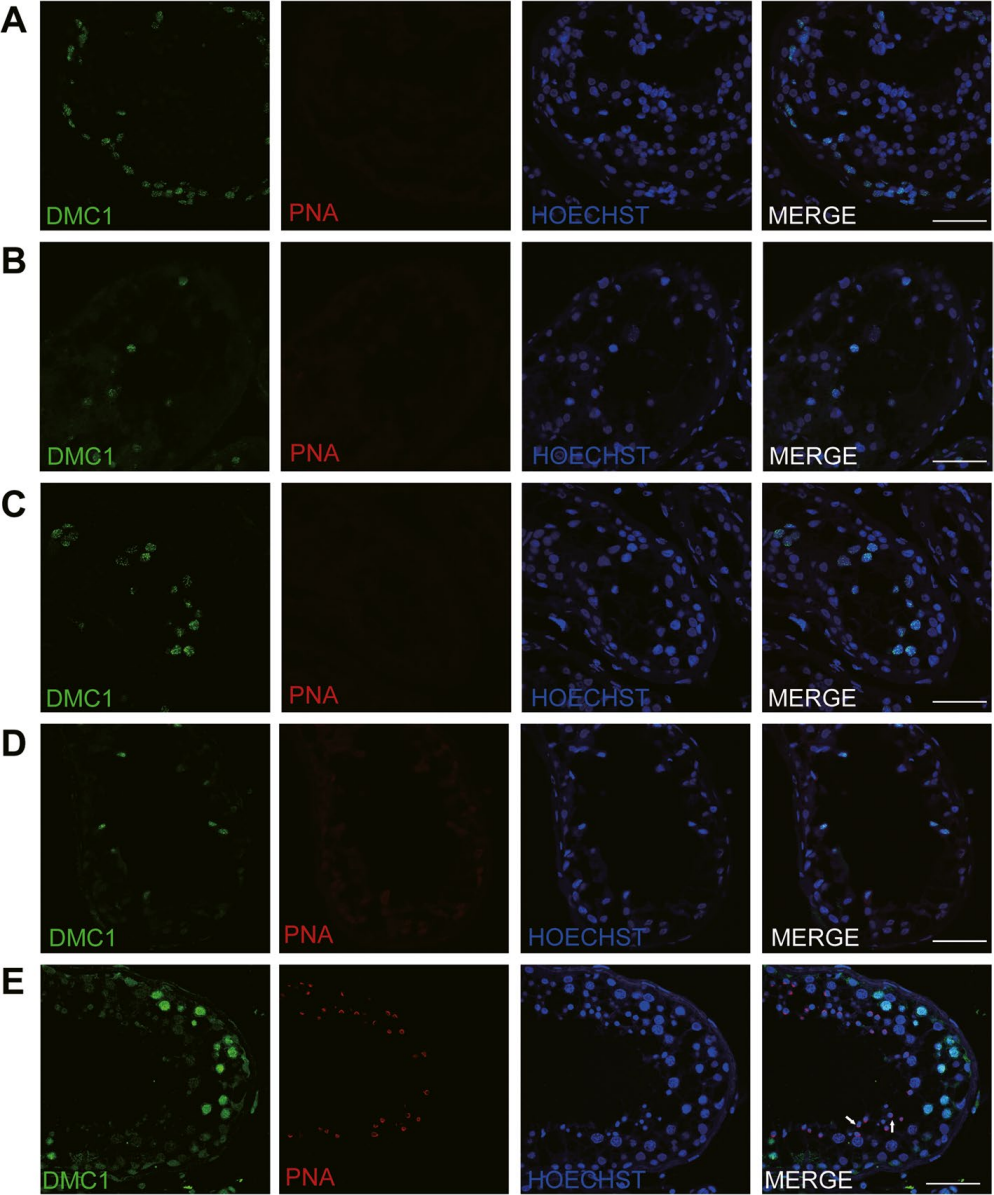
Expression of recombination proteins DMC1 and acrosomal marker PNA in the testis of patients with NOA and a patient with OA as a positive control. IHC staining showed the expression of DMC1 (green) and PNA (red) in the testis of the P9359 (**A**), P9517 (**B**), P9540 (**C**), P21504 (**D**), and a patient with OA (**E**). Arrow indicates the acrosome of spermatids in the testis. Scale bars = 50 μm

**Fig. 5:**
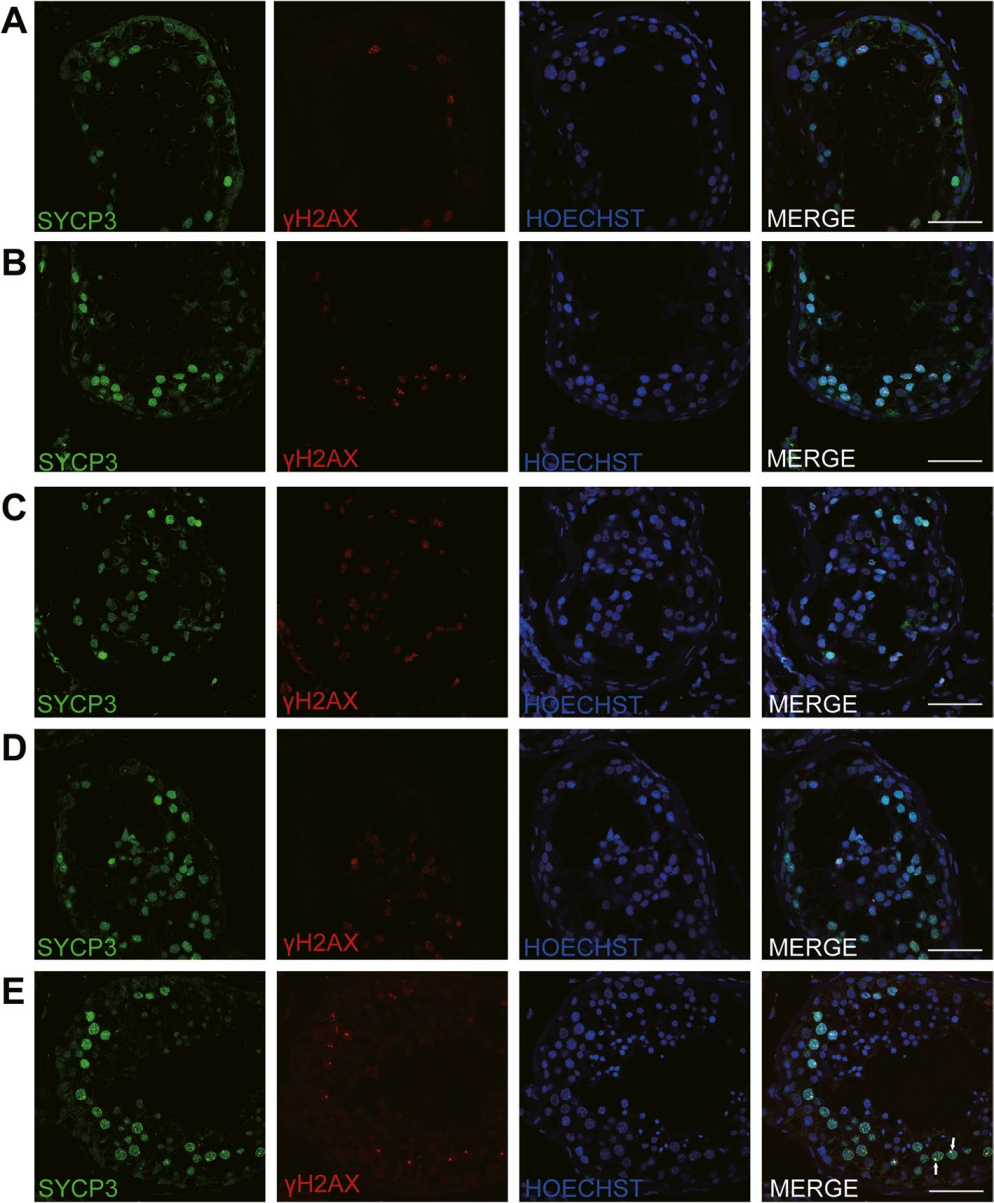
Expression of SYCP3 and γH2AX in the testis of NOA-affected patients and a patient with OA as positive control. IHC staining showed the expression of SYCP3 (green) and γH2AX (red) in the testis of the P9359 (**A**), P9517 (**B**), P9540 (**C**), P21504 (**D**), and a patient with OA (**E**). The arrow indicates the XY body in the spermatocytes in the testis. Scale bars = 50 μm

Four patients (P9517, P9540, P9359, P21504) received testicular biopsies, and no sperm was found. Compared with the testis of OA-affected patients (the positive control), the testicular histology from NOA-affected patients showed decreased number of spermatocytes and absence of spermatozoa and spermatids, but the number of spermatogonia and Sertoli cells at the basement membrane within the tubules remained unchanged (Fig. 3). IHC revealed the expression of DMC1 (indication for double-strand break repair) in the affected patients’ seminiferous tubules. However, there was no signal of PNA (a signal of acrosome), which is a marker of spermatids and spermatozoa (Fig. 4A-D). Furthermore, there were positive expressions of SYCP3 and γH2AX signal in the NOA-affected patients (Fig. 5A-D). SYCP3 was used to label components of the axial/lateral element. In preleptotene to zygotene spermatocytes, γH2AX staining showed pan-nuclear signal, whereas it could also label XY body, a visibly distinct domain in the nucleus of the pachytene spermatocytes. In the four NOA-affected patients, γH2AX was prominent in the preleptotene to zygotene spermatocytes, however, XY body was not detected in the biopsy (Fig. 5A-D). In contrary, the expression of SYCP3, γH2AX, DMC1 and PNA were positive in the OA’s testis (Figs. 4E and 5E). Altogether, the results indicated spermatocytes stage arrest for all the four NOA-affected patients with bi-allelic mutations in *MSH4*.

## Discussion

In the current study, six novel variants in *MSH4* were identified in the four NOA-affected patients, including two homozygous variants and two compound heterozygous variants. In silico analysis and functional assays revealed the pathogenicity of the variants in *MSH4*, suggesting these 6 novel variants could cause meiotic arrest and NOA.

NOA can be classified into Sertoli cell only syndrome, maturation arrest (MA), and hypospermatogenesis [14]. Uniform MA is a unique subset of NOA, and the incidence of genetic disorders in NOA patients with MA is higher than other types of NOA [15]. To date, several genes have been identified to be associated with meiosis defects in human via pedigree study, including *TEX11*(OMIM:300311), *SHOC1* (OMIM:618038), *TEX14* (OMIM: 605792), *STAG3* (OMIM: 608489), *DMC1* (OMIM: 602721), *SYCE1* (OMIM: 611486), *MSH5* (OMIM: 603382) and *MSH4* (OMIM: 602105) [16–22].

MSH4, a meiosis-specific gene located at 1p31.1 in human, has 20 exons encoding a 936-aa protein. MSH4 belongs to the DNA mismatch repair family of proteins. And MSH4 could form an obligate heterodimer with MutS homolog partner MSH5, which is essential in meiotic recombination. The loss of *Msh4* or *Msh5* in mice resulted in defects of prophase I progression, with almost complete failure of homologous synapsis [9–11]. To date, WES of pedigree studies reported only two types of homozygous mutation in *MSH4* (NM_002440.4: c.2261C > T and c.1552C > T) were associated with NOA [12, 13]. MutS heterodimers are characterized by five distinct MutS domains (I-V), and each domain is responsible for a specific function. MutS domain I recognizes DNA mismatches, and MutS domain II functions as a connector between MutS domain I and MutS domain III (the core). Furthermore, MutS domain V (the ATPase domain) contains the ABC responsible for conformational changes in the clamp structure. Collectively, MSH4-MSH5 dimer is essential for meiotic recombination.

In the current study, we reported that six novel *MSH4* variants were associated with meiotic arrest and NOA. The homozygous LoF mutations in *MSH4* (NM_002440.4: c.805_812del:p.V269Qfs*15; and NM_002440.4: c.2220_2223del:p.K741Rfs*2) led to a truncated MSH4 protein without expression of the ATP-binding domain, which has been demonstrated to be essential for MSH4 function. The compound heterozygous LoF mutations in *MSH4* (NM_002440.4: c.G1950A:p.W650X and c.2179delG: p.D727Mfs*11, respectively) were identified in family P9517. The allele frequency of maternal *MSH4* variant NM_002440.4: c.G1950A:p.W650X (rs149910287) was 8.3 × 10^− 6^ in the ExAC_All database, while the allele frequency of paternal MSH4 variant NM_002440.4: c.2179delG:p.D727Mfs*11 has not been reported before (https://gnomad.broadinstitute.org/). And these two variants resulted in the defect in expression of MutS V domain. In family P9540, a maternally inherited frameshift variant (NM_002440.4: c.670delT: p.L224Cfs*3) led to a truncated MSH4 protein without expression of MutS III-V domain and a paternally inherited variant (NM_002440.4: c.G244A: p.G82S) resulted in transition of Glycine to Serine at the position 82 of *MSH4*. And this missense variant was predicted to be potentially deleterious simultaneously by SIFT and MutationTaster.

In the current study, IHC facilitated to more accurately evaluate the period of spermatogenesis arrest. It was showed that DMC1 was expressed in all the testicular tissue with bi-allelic *MSH4* mutations, suggesting normal initiation of DSB repair in these patients. However, signals of PNA labeling spermatids and spermatozoa could not be detected in these biopsies. In normal spermatogenesis, γH2AX expression showed pan-nuclear pattern during preleptotene to zygotene stages, and in the pachytene stage, γH2AX-foci represented sex bodies. SYCP3 is a marker of the axial/lateral element (AE and LE). During the leptotene and zygotene, AE forms and extends, and in the pachytene stage, complete synapses form. However, there was no signals of XY body indicated by γH2AX staining in the seminiferous tubules of the patients with bi-allelic *MSH4* mutations. And SYCP3 exhibited the characteristics of the zygotene stage, suggesting the spermatogenesis arrested at zygotene in these patients with NOA.

In conclusion, we identified six novel mutations in the meiotic gene *MSH4* in four pedigrees with NOA. The meiotic arrest phenotype was confirmed in the NOA-affected patient. Further studies are required to identify the pathological role of the missense variant (NM_002440.4: c.G244A: p.G82S) and investigate the contribution of *MSH4* variants in infertile men in larger cohorts.

## Supplementary Information

**Additional file 1: Figure S1.** The conservation of missense mutation (p.G82S) in MSH4 protein. The conserved glycine amino acid at position 82 was changed to serine amino acid.

**Additional file 2: Table S1.** Primer sequences used for Sanger sequencing.

## Data Availability

The datasets used and analyzed during the current study are available from the corresponding author on reasonable request.

## Abbreviations

CO: Crossover
DSBs: Double-strand breaks
H&E: Hematoxylin and eosin staining
IHC: Immunohistochemistry
LoF: Loss of function
MA: Maturation arrest
NCO: Non-crossover
NOA: Non-obstructive azoospermia
OA: Obstructive azoospermia
PCR: Polymerase chain reaction
SC: Synaptonemal complex
SIFT: Sorting Intolerant From Tolerant
WES: Whole-exome sequencing
