# Supplementary figures and images for: Novel bi-allelic MSH4 variants causes meiotic arrest and non-obstructive azoospermia
Source: Reprod Biol Endocrinol. 2022 Jan 28;20:21. doi: 10.1186/s12958-022-00900-x (PMC8796546; doi:10.1186/s12958-022-00900-x)

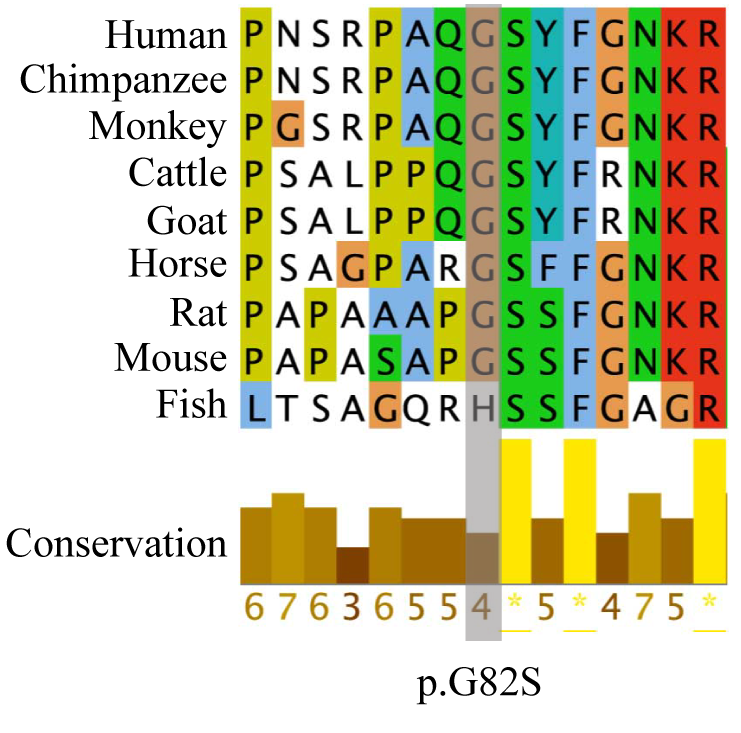

Supplement: Supplementary file 1 — Additional file 1: Figure S1. The conservation of missense mutation (p.G82S) in MSH4 protein. The conserved glycine amino acid at position 82 was changed to serine amino acid. [file 12958_2022_900_MOESM1_ESM.tif]
