# Supplementary material for: Novel bi-allelic MSH4 variants causes meiotic arrest and non-obstructive azoospermia
Source: Reprod Biol Endocrinol. 2022 Jan 28;20:21. doi: 10.1186/s12958-022-00900-x (PMC8796546; doi:10.1186/s12958-022-00900-x)
Supplement: Supplementary file 2 — Additional file 2: Table S1. Primer sequences used for Sanger sequencing. [file 12958_2022_900_MOESM2_ESM.docx]

**Table S1. Primer sequences used for Sanger sequencing**

| Patient No. | Change in Coding DNA (NM_0024401) | Forward primers (5’-3’) | Reverse primers (5’-3’) |
| --- | --- | --- | --- |
| P9359 | c.805_812del | CGCATGGCACTTCAAACTTTAT | TTACTCCTATGCCCTCACATTTC |
| P9517 | c.G1950A | GAGCTCAGGACAGTGGTAAAG | CTGGGCCATAATCTGACAAAGA |
| P9517 | c.2179delG | GAGTAGTTGGGACCACAGTTTC | ACTGCCCTCTGGCTCTTA |
| P9540 | c.G244A | GTCGCTCAGAAACCTCATACTT | ATCCCACCTTAGCAGGATTTG |
| P9540 | c.670delT | CCCTGGGCTCAAACTATTCTC | GACTCACTCATGCCTGTAATCC |
| P21504 | c.2220_2223del | GGACTGGGAAAGTTTAAGTGCTG | CCTACTTGACGTTTGCTCAATCA |
